# A Comprehensive Plasma Metabolomics Dataset for a Cohort of Mouse Knockouts within the International Mouse Phenotyping Consortium

**DOI:** 10.3390/metabo9050101

**Published:** 2019-05-22

**Authors:** Dinesh K. Barupal, Ying Zhang, Tong Shen, Sili Fan, Bryan S. Roberts, Patrick Fitzgerald, Benjamin Wancewicz, Luis Valdiviez, Gert Wohlgemuth, Gregory Byram, Ying Yng Choy, Bennett Haffner, Megan R. Showalter, Arpana Vaniya, Clayton S. Bloszies, Jacob S. Folz, Tobias Kind, Ann M. Flenniken, Colin McKerlie, Lauryl M. J. Nutter, Kent C. Lloyd, Oliver Fiehn

**Affiliations:** 1 NIH-West Coast Metabolomics Center, University of California, Davis, 451 Health Sciences Drive, Davis, CA 95616, USA; dinkumar@ucdavis.edu (D.K.B.); ythzhang@ucdavis.edu (Y.Z.); tsshen@ucdavis.edu (T.S.); slfan@ucdavis.edu (S.F.); brsroberts@ucdavis.edu (B.S.R.); psfitzgerald@ucdavis.edu (P.F.); bwance@ucdavis.edu (B.W.); lmvaldiviez@ucdavis.edu (L.V.); wohlgemuth@ucdavis.edu (G.W.); gsbyram@ucdavis.edu (G.B.); yychoy@ucdavis.edu (Y.Y.C.); bdhaffner@ucdavis.edu (B.H.); mshowalter@ucdavis.edu (M.R.S.); avaniya@ucdavis.edu (A.V.); csbloszies@ucdavis.edu (C.S.B.); jfolz@ucdavis.edu (J.S.F.); tkind@ucdavis.edu (T.K.); 2 The Centre for Phenogenomics, Toronto, ON M5T 3H7, Canada; flenniken@lunenfeld.ca (A.M.F); colin.mckerlie@sickkids.ca (C.M.); lauryl.nutter@sickkids.ca (L.M.J.N.); 3 Lunenfeld-Tanenbaum Research Institute, Mount Sinai Hospital, Toronto, ON M5G 1X5, Canada; 4 The Hospital for Sick Children, Toronto, ON M5G 1X8, Canada; 5 Mouse Biology Program, University of California, Davis, Davis, CA 95616, USA; KCLloyd@ucdavis.edu

**Keywords:** Metabolic phenotyping, metabolomics, lipidomics, functional genomics, mouse knockouts, IMPC, LC-MS, GC-MS

## Abstract

Mouse knockouts facilitate the study ofgene functions. Often, multiple abnormal phenotypes are induced when a gene is inactivated. The International Mouse Phenotyping Consortium (IMPC) has generated thousands of mouse knockouts and catalogued their phenotype data. We have acquired metabolomics data from 220 plasma samples from 30 unique mouse gene knockouts and corresponding wildtype mice from the IMPC. To acquire comprehensive metabolomics data, we have used liquid chromatography (LC) combined with mass spectrometry (MS) for detecting polar and lipophilic compounds in an untargeted approach. We have also used targeted methods to measure bile acids, steroids and oxylipins. In addition, we have used gas chromatography GC-TOFMS for measuring primary metabolites. The metabolomics dataset reports 832 unique structurally identified metabolites from 124 chemical classes as determined by ChemRICH software. The GCMS and LCMS raw data files, intermediate and finalized data matrices, R-Scripts, annotation databases, and extracted ion chromatograms are provided in this data descriptor. The dataset can be used for subsequent studies to link genetic variants with molecular mechanisms and phenotypes.

## 1. Summary

The human genome database has been instrumental to link cellular functions with genetic variants [1]. Yet, biological functions remain unclear for many genes, hampering their applications in clinical and translational approaches. Often, gene inactivation affects more than one biological function, known as gene pleiotropy. Similarly, genetic variants were also found to be associated with more than one phenotype in population level genome wide association studies (GWAS) [2,3,4,5]. GWAS catalogues such as the database of Genotypes and Phenotypes (dbGaP) started associating various phenotypes with genetic variants [6], but such associations lack causal relationships. Gene functions can be characterized on different biological levels from metabolite to cellular to whole-body phenotypes. 

Here, animal models help chart molecular pathways from genetic variant to phenotype [7]. The International Mouse Phenotyping Consortium (IMPC) is a network of centers with expertise in mouse genetics and phenotyping. The IMPC has established pipelines to generate knockout mice for over 7000 genes and aims to cover all 20,000 protein coding genes in mice [8,9]. The consortium has also identified mouse models for 360 diseases among the first 3328 mouse knockouts phenotyped [10]. The IMPC uses high throughput assays to measure phenotypes throughout the life of a knockout mouse and have successfully associated 974 genes with metabolic phenotypes and diseases [11]. Biomedical researchers can access IMPC services to receive specific knockout biospecimens and search associated phenotype data using the mousephenotype.org website. All the IMPC generated data are publicly available at http://www.mousephenotype.org.

Up to 10% of all human genes are involved in operation and regulation of metabolism [12] and it is well known that metabolism is dysregulated in many diseases. Several genes have well-characterized metabolic phenotypes that can be detailed by associating changes in metabolite levels (such as high cholesterol or low plasma uric acid) with genetic variants. Currently, the IMPC measures only a few metabolic endpoints such as body mass, plasma triglycerides, glucose tolerance, and basal blood glucose levels, warranting the need to expand their metabolic phenotype spectrum [11]. Over the past 20 years, metabolomics [13,14,15] has achieved an increased breadth and depth of analysis due to advances in sensitivity and accuracy of mass spectrometers, and up to 900 identified metabolites can be measured in blood plasma [16]. 

In this data descriptor, we provide a comprehensive metabolomics dataset and a phenotype dataset for plasma specimens of 30 mouse knockouts and their strain-matched wild type controls. Data were acquired by integrating three non-targeted assays (on primary metabolism, biogenic amines and complex lipids) with two targeted assays (oxylipins and combined bile acids and steroids), using both GC-TOFMS and different LC-MS protocols. 

## 2. Data Description

Raw GC-TOFMS and LC-MS mass spectra files are available at the NIH Metabolomics Workbench database (http://metabolomicsworkbench.org) (Accession number ST001154). Processed data matrices for all assays are provided in Table S11. The filtered metabolomics dataset is provided in the Table S12. Phenotype data for the mouse strains is provided at (Data citation 10). Data dictionary (Table S13), data matrix (Table S14), and sample metadata (Table S15) are provided in the supplementary section. Data file to sample label mapping is provided in the Table S10. The file also contains sample labels to IMPC accession IDs so metabolite to phenotype data can be linked. Analysis sequences for each assay are provided in Table S17 to check for batch effects or systematic error within the datasets. Annotation files are provided in the Tables S3–S7. Multiple reaction monitoring (MRM) transitions for the targeted assays are provided in Tables S8 and S9. Processed results for each assay are provided in the Supplemental Table S11. 

To ensure a high data quality dataset, the following strategies were adopted while analyzing these samples: (a) use of internal standards mixture, (b) analysis of quality control blood plasma samples; (c) analysis of blank samples to monitor carry-over and chemical artifacts including laboratory contaminants; (d) removal of multiple metabolite detections in different metabolomic platforms; (e) signal corrections using SERRF normalization for GC-TOFMS data, (f) removal of compounds with > 50% missing values, (g) removal of compounds with > 50% RSD technical variance, (h) use of curated annotation databases to form a target list for peak intensity data processing; and (i) mapping peaks with compound identifiers and SMILES code for informatics analyses. 

To show the technical reproducibility of the utilized LC-MS assays, RSD for peak heights of the internal standards were computed. Table 1 shows the RSD values for these standards. Median RSDs for the detected compounds were 8% (GCMS), 11.5% (CSH-POS), 13% (CSH-NEG), 12% (HILIC-POS), and 52% (HILIC-NEG). No batch effect was observed from the HILIC-POS, CSH-POS, and CSH-NEG datasets. For HILIC-NEG, four batches were observed, and the signals were corrected using the median-batch normalization, dividing the value for each metabolite by its median value within a batch. 

Targeted assays utilized a ten-point calibration curve to calculate molar concentrations of the target analytes. Values that did not pass the limit of quantification were not included in the data matrix, leading to many missing values. 

## 3. Methods

### 3.1. IMPC Consortium, Mouse Knockout Selection and Plasma Samples

The International Mouse Phenotyping Consortium (www.mousephenotype.org) provided blood plasma samples for 30 knockout strains (Table 2). For each knockout line, three male and three female mice were selected, and a total of 40 C57BL/6NCrl baseline control wild-type mice were used to match the knockout strains. Plasma samples were shipped to the West Coast Metabolomics Center (WCMC; http://metabolomics.ucdavis.edu) on dry ice. Samples were stored at −80 °C until analyzed. Each sample was assigned a unique identifier according to the sampling date and time at The Centre for Phenogenomics (TCP) (See Table S15). Twenty additional human pool plasma samples (BioIVT, previously known as BioreclamationIVT) and up to 10 method blanks were analyzed along with the mouse plasma samples for each analytical assay. Mouse knockout were selected if (1) plasma sample for a knockout was already available at IMPC (2) PubMed literature searches for the gene yielded some papers in reference to metabolism and (3) gene assayed in an IMPC proteomics assay.

All experimental procedures on animals received approval from the Animal Care Committee of The Centre for Phenogenomics and were conducted in accordance with the guidelines of the Canadian Council on Animal Care. TCP’s approved lincense numbers are - Animal Use Protocol (AUP) 0153, 0275, 0277, 0279. Additionally, all animal production followed the Animal Research: Reporting of in vivo Experiments (ARRIVE) guidelines within the context of the International Mouse Phenotyping Consortium (IMPC). The human plasma samples were commercially acquired from BioIVT and their use was approved by the Independent Institutional Review Board, Florida. The study identification number for BioIVT plasma samples is 201209942.

### 3.2. Metabolomics Facility

**Figure 1:**
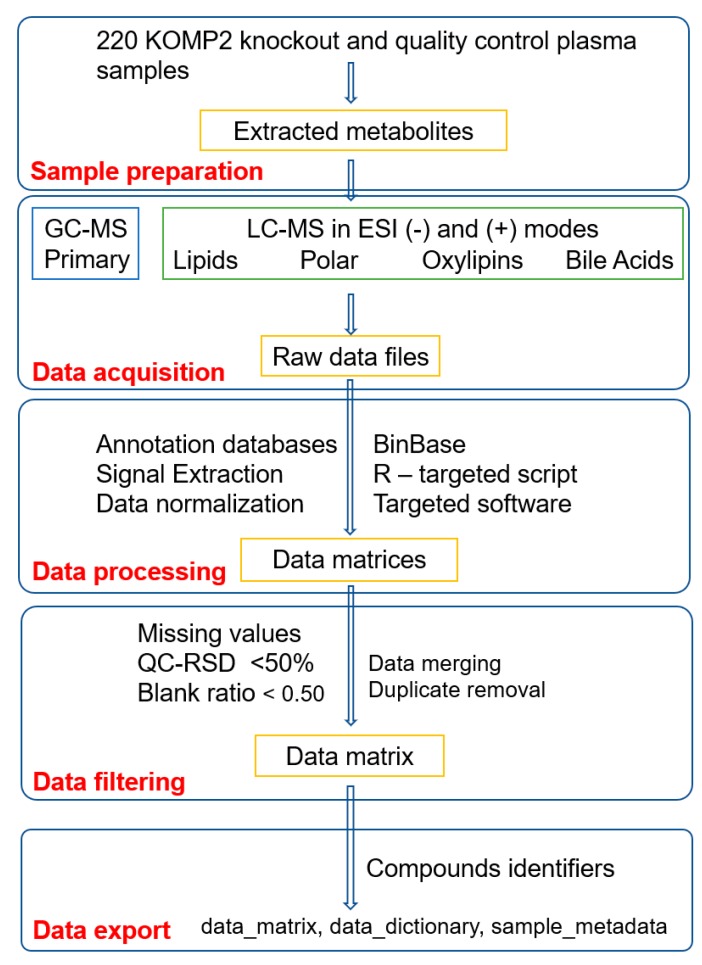
Overview of the metabolomics data generation and quality control workflow for 220 knockout mouse plasma (KOMP2) samples. A less stringent relative standard deviation (RSD) and sample to blank ratio were used because the effect size of two or more show a major effect. As raw spectra files are provided for this study, a user can re-generate the data matrix with different thresholds. Abbreviation: GCMS—gas chromatography and mass spectrometry, LCMS—liquid chromatography and mass spectrometry and ESI—electrospray ionization.

Metabolomics data for the mouse plasma were acquired using seven analytical assays using GC-MS and LC-MS platforms (Table 1). All LC-MS methods were performed using electrospray ionization (ESI). These assays are routinely used to generate metabolomics data at the WCMC for almost 30,000 samples per year, including many blood samples [13,15,17,18]. The WCMC use large and validated lists of metabolite targets (Tables S3–S9), large mass spectral libraries from the MassBank of North America (MoNA available at http://massbank.us) to annotate novel compounds, standardized samples preparation and data acquisition methods, robust data processing using freely available MS-DIAL [19], SERRF software [20], the BinBase mass spectral database [21] for covering over 150,000 GC-TOFMS samples analyzed over the past 15 years, and a variety of data analysis and interpretation tools, including statistics [22], pathway and network mapping [23], and metabolite enrichment analysis [24]. Figure 1 shows the overview of the metabolomics data generation and quality control workflow.

### 3.3. Annotation Databases for Untargeted Metabolomics

#### 3.3.1. Gas Chromatography and Mass Spectrometry

Every acquired GC-TOFMS spectrum for blood specimens has been stored in the BinBase database for past 15 years at the WCMC. The database contain over 150,000 samples which can be queried through the BinVestigate web GUI (https://binvestigate.fiehnlab.ucdavis.edu/#/) for identified or unknown metabolites that are confidently detected in over 100 tissues and species [21]. The BinBase algorithm [25] utilizes this annotation database to generate a raw result data matrix (Table S11). The current BinBase annotation database is provided in supplementary Table S3 with 1205 annotated spectra for 588 unique compounds detected in biological samples. 

#### 3.3.2. Hydrophilic Interaction Liquid Chromatography (HILIC) Mass Spectrometry

A database of target metabolites detected in HILIC-ESI-MS using both positive or negative electrospray mode are provided in supplementary Tables S4 and S5. This target database was generated by searching MS/MS spectra for blood specimens acquired in past three years against the NIST17 MS/MS, the LipidBLAST [26] and MoNA databases, in addition to a specific HILIC-retention time MS/MS mass spectral library of 1200 authentic standards [27]. For negative ESI mode, the HILIC-NEG annotation database yielded 107 identified compounds in the mouse plasma data set presented here using mass-to-charge (*m*/*z*), retention time (RT), and fragmentation spectra (MS/MS) match. An additional 45 compounds were annotated by *m*/*z* and MS/MS fragmentation matches and one compound was annotated by *m*/*z* and RT match. The abundance of this one compound was too low to trigger an experimental MS/MS event in data dependent MS/MS data acquisition methods. For the positive ESI mode, the HILIC-POS annotation database of the mouse plasma dataset presented here yielded 84 compounds that were annotated by *m*/*z*, RT, and MS/MS matching, 86 compounds annotated by *m*/*z,* and MS/MS data only, and 28 compounds were annotated by *m*/*z* and RT match.

#### 3.3.3. Charged Surface Hybrid Liquid Chromatography (CSH) and Mass Spectrometry

The CSH database for target mouse plasma lipids for positive and negative electrospray modes is provided in the supplementary Tables S6 and S7. The database is generated by searching MS/MS spectra for blood specimens acquired in past seven years against NIST17 MS/MS database and LipidBLAST mass spectral libraries. The CSH-NEG annotation database contains 215 verified lipids with m/z and MS/MS match; the CSH-POS annotation database contains 304 compounds with validated m/z and MS/MS match. 

### 3.4. Assay 1. Gas Chromatography and Mass Spectrometry

#### 3.4.1. Sample Preparation

One milliliter of degassed, −20 °C cold solvent mixture of acetonitrile (ACN):isopropanol (IPA):water (H_2_O) (3:3:2, *v*/*v*/*v*) was added to each 20 µL mouse plasma aliquot. Samples were vortexed for 10 seconds, shaken for 5 min and then centrifuged for 2 min at 14,000 rcf (relative centrifugal force). Two 450 µL supernatant aliquots were transferred to new tubes. To remove any excess protein, the supernatant was extracted with 500 µL 1:1 acetonitrile:water and vortexed for 10 seconds, centrifuged for 2 min at 14,000 rcf. The supernatant was transferred to a clean tube and then dried down in a CentriVap concentrator. For derivatization, 10 µL of methoxyamine hydrochloride in pyridine (40 mg/mL) was added to each sample and then shaken at 30 °C for 90 min. Then 90 µL of *N*-methyl-*N*-(trimethylsilyl) trifluoroacetamide (MSTFA, Sigma-Aldrich) was added for trimethylsilylation. C8–C30 fatty acid methyl esters (FAMEs) were added as internal standard (See Supplementary Table S18) for retention time correction. Samples were shaken for 30 min at 37 °C. These derivatized samples were analyzed by GC-MS using a Leco Pegasus IV time of flight mass spectrometer. For more details see [28].

#### 3.4.2. Data Acquisition

An Agilent 6890 gas chromatography instrument equipped with a Gerstel automatic linear exchange systems (ALEX) which included a multipurpose sample dual rail and a Gerstel cold injection system (CIS). The CIS temperature program was: 50 °C to 275 °C final temperature at a rate of 12 °C/s and held for 3 min. Injection volume was 0.5 µL with 10 µL/s injection speed. Injection mode was splitless with a purge time of 25 seconds. Injector liner was changed after every 10 samples. Injection syringe was washed with 10 µL of ethyl acetate before and after each run. A Rtx-5Sil MS column (30 m length, 0.25 mm i.d., 0.25 microM 95% dimethyl 5% diphenyl polysiloxane film). An additional 10 m integrated guard column was used. Mobile phase was 99.9999% pure Helium gas with a flow rate of 1 mL/min. GC temperature program was: held at 50 °C for 1 min, ramped at 20 °C/min to 330 °C and then held for 5 min. A Leco Pegasus IV time of flight mass spectrometer was used to acquire data. The transfer line temperature between gas chromatograph and mass spectrometer was set to 280 °C. Electron ionization at −70 V was employed with an ion-source temperature of 250 °C. Acquisition rate was 17 spectra/second with a scan mass range of 85–500 Dalton (Da).

#### 3.4.3. Data Processing

Raw GC-TOF MS data files were preprocessed directly after data acquisition and stored as ChromaTOF-specific peg files, as generic txt result files and additionally as generic ANDI MS cdf files. ChromaTOF version 4.0 was used for data preprocessing without smoothing, 3 s peak width, baseline subtraction just above the noise level, and automatic mass spectral deconvolution and peak detection at signal/noise (s/n) levels of 5:1 throughout the chromatogram. Results in .txt format were exported to a data server with absolute spectra intensities and further processed by a filtering algorithm implemented in the metabolomics BinBase database. The BinBase algorithm (rtx5) used the following settings: validity of chromatogram (10^7^ counts/s), unbiased retention index marker detection (MS similarity > 800, validity of intensity range for high *m/z* marker ions), retention index calculation by 5th order polynomial regression. Spectra were cut to 5% base peak abundance and matched to database entries from most to least abundant spectra using the following matching filters: retention index window ±2000 units (equivalent to about ±2 s retention time), validation of unique ions and apex masses (unique ion must be included in apexing masses and present at >3% of base peak abundance), mass spectrum similarity must fit criteria dependent on peak purity and signal/noise ratios and a final isomer filter. Failed spectra were automatically entered as new database entries if signal/noise ratios were larger than 25 and mass spectral purity better than 80%. All thresholds reflect settings for ChromaTOF v. 4.0. Quantification was reported as peak height using the unique ion as default, unless a different quantification ion was manually set in the BinBase administration software BinView. A quantification report table was produced for all database entries that were positively detected in more than 10% of the samples of this mouse knockout study. A subsequent post-processing module was employed to automatically replace missing values from the .cdf files. Prior to statistical analyses, data were filtered by combining multiple signals associated with each unique metabolite due to derivatization reactions. All metabolic signals were discarded if *s*/*n* > 3 in comparison to blanks, or if replaced values were >3 the intensity of truly detected values. Data were normalized using a random forest algorithm-based signal correction method [20] available at (http://serrf.fiehnlab.ucdavus.edu).

### 3.5. Assay 2 and 3. Hydrophilic Interaction Liquid Chromatography (HILIC) Q-Exactive HF Mass Spectrometry for Polar Metabolites

#### 3.5.1. Sample Preparation 

Metabolites were extracted from 20 µL of mouse plasma using 1 mL of degassed, −20 °C cold mixture of ACN:IPA:H_2_O (3:3:2, *v*/*v*/*v*). Samples were vortexed for 10 seconds, shaken for 5 min and then centrifuged for 2 min at 14,000 rcf. Two 450 µL supernatant aliquots were transferred to new tubes. One tube was stored as a backup aliquot and another was dried in a SpeedVac concentrator. Sample were re-suspended with 100 µL of ACN:H_2_O (80:20, *v*/*v*) which contained deuterium labeled internal standards (See Supplementary Table S18) prior to injection. 

#### 3.5.2. Data Acquisition

3 µL sample aliquots were injected on a Waters Acquity UPLC BEH Amide column (150 mm length × 2.1 mm i.d.; 1.7 μm particle size) maintained at 45 °C. A Waters Acquity VanGuard BEH Amide pre-column (5 mm × 2.1 mm i.d.; 1.7 μm particle size) was used as guard column. Mobile phase A was 100% LC-MS grade H_2_O with 10 mM ammonium formate and 0.125% formic acid and mobile phase B was 95:5 *v*/*v* ACN:H_2_O with 10 mM ammonium formate and 0.125% formic acid. Gradient was started at 100% (B) for 2 min, 70% (B) at 7.7 min, 40% (B) at 9.5 min, 30% (B) at 10.25 min, 100% (B) at 12.75 min, and isocratic until 16.75 min. The column flow was 0.4 mL/min. Vanquish UHPLC system (ThermoFisher Scientific) was used. A Thermo Q-Exactive HF Orbitrap MS instrument was operated in positive and negative ESI mdoes respectively with the following parameters: mass range 60−900 *m/z*; spray voltage 3.6kV (ESI+) and −3kV (ESI−), sheath gas (nitrogen) flow rate 60 units; auxiliary gas (nitrogen) flow rate 25 units, capillary temperature 320 °C, full scan MS1 mass resolving power 60,000, data-dependent MSMS (dd-MSMS) 4 scans per cycle, normalized collision energy at 20%, 30%, and 40%, dd-MSMS mass resolving power 15,000. Thermo Xcalibur 4.0.27.19 was used for data acquisition and analysis. Instruments was tuned and calibrated by manufacturer’s recommendations.

#### 3.5.3. Data Processing

Raw data files were converted to the mzML format using the ProteoWizard MSConvert utility. For each *m/z* values ion chromatogram was extracted with *m/z* thresholds of 0.005 Da and retention time threshold of 0.10 min. Apex of the extracted ion chromatograph was used as peak height value and exported to a text file. Peak height files for all the samples were merged together to generate a data matrix. Targeted peak height signal extraction was performed using an R script which is provided at the GitHub repository (https://github.com/barupal). HILIC-POS data were not normalized because no batch effect was observed (Supplementary Figure S1). HILIC-NEG data were normalized by the median value for each batch to remove batch effects.

### 3.6. Assay 4 and 5. CSH-C18 Q-Exactive HF Mass Spectrometry for Lipidomics

#### 3.6.1. Sample Preparation

Lipids were extracted from a 20 µL aliquot of plasma. 225 µL of cold methanol (MeOH) containing a mixture of deuterated lipid internal standards (See Supplementary Table S18) was added and samples were vortexed for 10 s. Then 750 µL of methyl tertiary-butyl ether (MTBE) was added. Samples were vortexed for 10 s and shaken for 5 min at 4 °C. Next, 188 µL water was added and samples were vortexed for 10 s and centrifuged for 2 min at 14000 rcf. Two 350 µL aliquots from the non-polar layer were prepared. One aliquot was stored at −20 °C as a backup and the other was evaporated to dry in a SpeedVac. Dried extracts were resuspended using a mixture of methanol/toluene (9:1, *v*/*v*) (60 µL) containing an internal standard [12-[(cyclohexylamino) carbonyl]amino]-dodecanoic acid (CUDA)] used as a quality control. Method blanks and pooled human plasma (BioIVT) were prepared along with the study samples for monitoring the data quality.

#### 3.6.2. Data Acquisition

Extracted lipids were separated on an Acquity UPLC CSH C18 column (100 × 2.1 mm; 1.7 µm) maintained at 65 °C. The mobile phases for positive mode consisted of 60:40 ACN:H_2_O with 10 mM ammonium formate and 0.1% formic acid (A) and 90:10 IPA:ACN with 10 mM ammonium formate and 0.1% formic acid (B). For negative mode, the mobile phase modifier was 10 mM ammonium acetate instead. The gradient was as follows: 0 min 85% (A); 0–2 min 70% (A); 2–2.5 min 52% (A); 2.5–11 min 18% (A); 11–11.5 min 1% (A); 11.5–12 min 1% (A); 12–12.1 min 85% (A); and 12.1–15 min 85% (A). Sample temperature is maintained at 4 °C in the autosampler. 2 µL of sample was injected. Vanquish UHPLC system (ThermoFisher Scientific) was used. Thermo Q-Exactive HF Orbitrap MS instrument was operated in both positive and negative ESI modes respectively with the following parameters: mass range 120−1700 *m/z*; spray voltage 3.6kV (ESI+) and −3kV (ESI−), sheath gas (nitrogen) flow rate 60 units; auxiliary gas (nitrogen) flow rate 25 units, capillary temperature 320 °C, full scan MS1 mass resolving power 60,000, data-dependent MS/MS (dd-MS/MS) 4 scans per cycle, normalized collision energy at 20%, 30%, and 40%, dd-MS/MS mass resolving power 15,000. Thermo Xcalibur 4.0.27.19 was used for data acquisition and analysis. The instrument was tuned and calibrated according to the manufacturer’s recommendations.

#### 3.6.3. Data Processing

Raw data files were converted to the mzML format using the ProteoWizard MSConvert utility. For each m/z values ion chromatogram was extracted with *m*/*z* thresholds of 0.005 Da and retention time threshold of 0.10 min. Apex of the extracted ion chromatograph was used as peak height value and exported to a txt file. Peak height files for all the samples were merged together to generate a data matrix. Targeted peak height signal extraction was performed using an R script that is available at https://github.com/barupal. Extracted ion chromatograms for each peak were saved as pictures. CSH-POS and CSH-NEG data matrices were generated. No normalization was applied as minimum signal drift was observed during analysis (Supplementary Figure S1). 

### 3.7. Assay 6 and 7. Bile Acids-Steroids, and Oxylipin Targeted Analysis

#### 3.7.1. Sample Preparation

After thawing on ice and vortexing, 50 µL of plasma from each sample was aliquoted to a polypropylene 96-well plate for extraction. The samples were spiked with internal standards of bile acids, steroids, and oxylipins at a concentration of 250 nM, resulting in a final concentration of 25 nM prior to LC-MS analysis. The suspensions were treated with antioxidant (0.2 mL/mL butylated hydroxytoluene and ethylenediaminetetraacetic acid (EDTA)). 10 µL of 1000 nM 1-cyclohexyluriedo-3-dodecanoic acid (CUDA) and 1-Phenyl 3-Hexadecanoic Acid Urea (PHAU) were added as quality markers for the analysis. ACN:MeOH 1:1 (*v*/*v*) were added to final volume of 250 µL. The samples were vortexed and incubated at 20°C for 30 min to precipitate protein. After centrifugation at 15,000 rcf for 5 min, the supernatant was transferred to a 0.2 µm PVDF filter plate (polyvinylidene fluoride membrane, Agilent). The solutions were collected in new polypropylene 96-well plates and stored at −20 °C until analyzed. 

#### 3.7.2. Data Acquisition

For bile acids and steroids, reverse-phase liquid chromatography was performed on a Waters Acquity BEH C18 column (1.7 µm, 2.1 × 100 mm) with its corresponding Vanguard precolumn at 45 °C at a flow rate of 400 µL/min. Mobile phase A was LC-MS grade H_2_O with 0.1% formic acid; mobile phase B was ACN with 0.1% formic acid. The 20 min gradient is: 0–0.5 min 10% B, 0.5–1 min 10–20% B, 1–1.5 min 20–22.5% B, 1.5–11 min 22.5–45% B, 11–12.5 min 45–95% B, 12.5–16 min 95% B, 16–16.5 min 95–10% B, 16.5–20 min 10% B.

For oxylipins, LC separation was conducted on the same column, but mobile phase A was H_2_O with 0.1% acetic acid and B was ACN:IPA 90:10 (*v*/*v*) with 0.1% acetic acid. The column was maintained at 45 °C at the flow rate of 250 µL/min. A 16 min gradient was used with 0–1 min gradient from 25–40% B, 1–2.5 min 40–42% B, 2.5–4.5 min 42–50% B, 4.5–10.5 min 50–65% B, 10.5–12.5 min 65–75% B, 12.5–14 min 75–85% B, 14–14.5 min 85–95% B, 14.5–15 min 95–25% B, 15–16 min 25% B.

Extracts were analyzed by liquid chromatography (Waters ACQUITY UPLC I-Class system) coupled to a Sciex 6500+ QTRAP hybrid, triple quadrupole linear ion trap mass spectrometer. 5 µL of each extract was injected. Scheduled multiple reaction monitoring (MRM) was performed with optimized collision energies, de-clustering potentials, and collision cell exit potentials for individual analyte. A LC-MRM targeted method was used to analyze both bile acids and steroids with positive and negative polarity switching. Oxylipins were analyzed in another LC-MRM method in negative ionization mode only. All analytes were quantified against 6-point calibration curves using internal standards. Turbo Spray Ion Source parameters are: curtain gas (CUR) 25 psi, nebulizer gas (GS1) 50 psi, turbo-gas (GS2) 50 psi, electrospray voltage −4.5 kV/+3 kV, and source temperature 525 °C. Nitrogen was used as the collision gas. Software Analyst 1.6.3 and MultiQuant 3.0.2 (AB Sciex) were used for data acquisition and quantification. MRM transitions for the analytes are provided in the supplementary Tables S8 and S9. 

#### 3.7.3. Data Processing

MultiQuant version 3.0.2 was used for the peak integration and peak area computation. Peak integration settings were: Gaussian smooth width at 1.0 points, retention half window at 10–15 s, updated expected RT checkbox ‘NO’, minimum peak width at 8 points, minimum peak height at 750, noise at 40%, baseline subwindow at 1.7 min and peak splitting at 3 points. Multi-Quant software was also used for computing the molar concentrations for the analytes by using calibration curves created using internal standards as described in the supplementary file (Tables S8 and S9).

### 3.8. Data Merging and Filtering

Data matrices from each platform were combined to generate a joint dataset for all the samples. It contained a total of 1215 signals of identified metabolites (Table S12). Afterwards, signals were retained if relative standard deviation (RSD) was better than 50% and if fewer than 50% missing values were observed (Table S13). The median RSD for compounds in QC sample was less than 20% for all assays except the HILIC-NEG mode data. Overall, up to 70% compounds have a QC RSD of less than 20% across all assays. A majority of labelled internal standards showed a relative standard deviation of less than 20% in LC-MS assays (Table 3). We also justify that for gene knockout experiments, investigators are usually interested in two or more folds effect sizes, so a 50% threshold should not compromise the statistical power if large effect sizes are sought. For metabolites that were detected in multiple platforms, data with the lowest relative standard deviation in the quality control samples were retained. The filtered dataset had 832 metabolites (Table S14). The simplified molecular-input line-entry system (SMILES) codes for all annotated lipids were obtained from the LipidBlast MSP file or from the PubChem Compound Identifier Exchange service (https://pubchem.ncbi.nlm.nih.gov/idexchange/idexchange.cgi) and provided in the data dictionary (Table S13). Chemical classes for the identified compounds were estimated using the ChemRICH software. Sample metadata is provided in the Table S15.

### 3.9. Phenotype Dataset

The phenotype dataset for each mouse knockout strainwas downloaded from the IMPC database (www.mousephenotype.org) using their R-package IMPCData. First, allele accession numbers were matched to the IMPC database identifiers. Then, for each mouse accession, phenotype data were retrieved using the mouse strain identifier and phenotype identifiers (Table S1). The overall phenotype dataset is provided in the Table S2. 

## 4. User Notes

Users can utilize raw spectra files, processed results, and the merged metabolomics dataset for the integration of phenotype and metabolomics dataset for each knockout strain. Raw spectra files should be used to check the quality of detected peaks and to annotate unknown metabolites with new mass spectral libraries. Raw data files can be converted to mzML format for importing in other software such as mzR or MZ-Mine. Proper data transformation and scaling for each data matrix from the assays is recommended before performing univariate and multi-variate statistical analysis. The dataset is particularly interesting for researchers who focus on the biological functions of the 30 genes studied here, specifically, their potential roles in metabolism. We performed a ChemRICH class annotation for the structurally identified compounds and found that almost 80 chemical classes werecovered. These chemical groups can be associated with genes and with phenotypes. We foresee this dataset’s use in developing next generation bioinformatics as well as in teaching courses for metabolomics and as a test case for benchmarking software. As we have provided the annotation database, mass spectral libraries and protocol details, these resources can be used to re-create similar datasets for other cohorts of the blood plasma specimens. 

## Figures and Tables

**Table 1:** Overview of the analytical assays.

Assays	Chromatography		Mass Spectrometer	Data Processing
	Column	Instrument		
Assay 1: - Primary	Rtx-5Sil MS column (30 m length, 0.25 mm i.d., 0.25 microM 95% dimethyl 5% diphenyl polysiloxane film)	Agilent 6890 GC	Leco GCTOF Pegasus IV	ChromaTOF 4/BinBase
Assay 2 and 3: - Polar (ESI + and ESI − )	Waters Acquity UPLC BEH Amide column (150 mm length × 2.1 mm i.d.; 1.7 μm particle size)	Thermo Vanquish UHPLC	Thermo Q-Exactive HF Orbitrap	NIST MS Search and R-target search
Assay 4 and 5: - Lipids (ESI − and ESI +)	Waters Acquity UPLC CSH C18 column (100 × 2.1 mm; 1.7 µm)	Thermo Vanquish UHPLC	Thermo Q-Exactive HF Orbitrap	NIST MS Search and R-target search
Assay 6 and 7: - Bile acids/steroids and Oxylipins	Waters Acquity BEH C18 column (1.7 µm, 2.1 mm × 100 mm)	Waters ACQUITY UPLC I-Class system	Sciex 6500+ QTRAP hybrid	MultiQuant 3.0.2 (AB Sciex)

**Table 2:** Details of the mouse strains.

NCBI Gene ID	Gene Symbol	IMPC Line	Gene Description
235661	Dync1li1	K2P2	Dynein Cytoplasmic 1 Light Intermediate Chain 1
71742	Ulk3	K2P2	unc-51-like kinase 3
14380	G6pd2	KOMP2	Glucose 6-phosphate dehydrogenase 2
29875	Iqgap1	KOMP2	IQ motif containing GTPase activating protein 1
23980	Pebp1	KOMP2	phosphatidylethanolamine binding protein 1
30939	Pttg1	KOMP2	pituitary tumor-transforming gene 1
11947	Atp5b	NorCOMM2	ATP synthase, H+ transporting mitochondrial F1 complex, beta subunit
11972	Atp6v0d1	NorCOMM2	ATPase H+ Transporting lysosomal V0 Subunit D1
12567	Cdk4	NorCOMM2	Cyclin Dependent Kinase 4
13361	Dhfr	NorCOMM2	Dihydrofolate reductase
68421	Lmbrd1	NorCOMM2	LMBR1 domain containing 1
18005	Nek2	NorCOMM2	NIMA (never in mitosis gene a)-related expressed kinase 2
67963	Npc2	NorCOMM2	NPC intracellular cholesterol transporter 2
19193	Pipox	NorCOMM2	Pipecolic acid oxidase
19877	Rock1	NorCOMM2	Rho-associated coiled-coil containing protein kinase 1
269378	Ahcy	NorCOMM2	S-adenosylhomocysteine hydrolase
232345	A2m	NorCOMM2	alpha-2-macroglobulin
230558	C8a	NorCOMM2	complement component 8, alpha polypeptide
14420	Galc	NorCOMM2	galactosylceramidase
26384	Gnpda1	NorCOMM2	glucosamine-6-phosphate deaminase 1
15926	Idh1	NorCOMM2	isocitrate dehydrogenase
67096	Mmachc	NorCOMM2	methylmalonic aciduria cblC type, with homocystinuria
17855	Mvk	NorCOMM2	mevalonate kinase
76293	Mfap4	NorCOMM2	microfibrillar-associated protein 4
54128	Pmm2	NorCOMM2	phosphomannomutase 2
16922	Phyh	NorCOMM2	phytanoyl- CoA hydroxylase
18817	Plk1	NorCOMM2	polo-like kinase 1, serine/threonine protein kinase
19248	Ptpn12	NorCOMM2	protein tyrosine phosphatase, non-receptor type 12
24068	Sra1	NorCOMM2	steroid receptor RNA activator 1
22631	Ywhaz	NorCOMM2	tyrosine 3-monooxygenase/tryptophan 5-monooxygenase activation protein

**Table 3:** Relative standard deviation of labelled internal standards for the liquid chromatography/ mass spectrometry (LC/MS) assays.

Assay	Internal Standard Name	*m*/*z* Value	Retention Time (min)	Relative Standard Deviation
CSHNEG	FA (16:0)-d3	258.2515	2.3	8%
CSHNEG	CUDA iSTD	339.2653	0.5	10%
CSHNEG	MAG (17:0/0:0/0:0)	403.3066	3.0	9%
CSHNEG	LPE (17:1)	464.2782	1.2	17%
CSHNEG	LPC (17:0)	568.362	1.7	8%
CSHNEG	Ceramide (d18:1/17:0)	610.5416	5.9	14%
CSHNEG	PC (12:0/13:0)	694.4665	3.5	8%
CSHNEG	PE (17:0/17:0)	718.5392	6.2	11%
CSHNEG	PG (17:0/17:0)	749.5338	4.9	14%
CSHNEG	SM (d18:1/17:0)	775.5971	5.3	52%
CSHPOS	LPC(17:0)	510.3554	1.7	5%
CSHPOS	PC(12:0/13:0)	636.4599	3.5	6%
CSHPOS	Cer(d18:1/17:0)	552.535	5.8	7%
CSHPOS	SM(d18:1/17:0)	717.5905	5.0	7%
CSHPOS	PE(17:0/17:0)	720.5538	6.2	7%
CSHPOS	CUDA	341.2799	0.7	8%
CSHPOS	LPE(17:1)	466.2928	1.2	8%
CSHPOS	MG(17:0/0:0/0:0)	367.2819	3.0	9%
CSHPOS	CE(22:1)	729.652	11.5	10%
CSHPOS	DG(12:0/12:0/0:0)	474.4153	4.2	12%
CSHPOS	Cholesterol d7	376.3955	4.7	12%
CSHPOS	DG(18:1/2:0/0:0)	416.3371	3.2	17%
CSHPOS	TAG d5(17:0/17:1/17:0)	874.7882	10.9	20%
CSHPOS	Sphingosine(d17:1)	286.2741	1.1	21%
HILICNEG	15N2-l-Arginine	175.0974	9.41	22%
HILICNEG	CUDA	339.2642	1.1	11%
HILICNEG	D3-Creatinine	115.0694	4.71	12%
HILICNEG	D3-dl-Alanine	91.0581	7.97	19%
HILICNEG	D3-dl-Aspartic acid	135.048	9.09	27%
HILICNEG	D3-dl-Glutamic acid	149.0636	8.65	27%
HILICNEG	D5-l-Glutamine	150.0922	8.46	20%
HILICNEG	Val-Tyr-Val	378.2023	6.79	9%
HILICPOS	15N2-l-Arginine	177.113	9.53	9%
HILICPOS	CUDA	341.2799	1.16	11%
HILICPOS	D3-1-Methylnicotinamide	140.0898	6.25	5%
HILICPOS	D3-AC(2:0)	207.1419	7.21	7%
HILICPOS	D3-Creatine	135.0956	8.15	9%
HILICPOS	D3-Creatinine	117.085	4.95	4%
HILICPOS	D3-dl-Alanine	93.0738	8.17	8%
HILICPOS	D3-dl-Aspartic acid	137.0636	9.34	9%
HILICPOS	D3-dl-Glutamic acid	151.0793	8.85	7%
HILICPOS	D3-Histamine, N-methyl-	129.1214	7.35	20%
HILICPOS	D3-l-Carnitine	165.1313	7.83	6%
HILICPOS	D5-l-Glutamine	152.1078	8.67	11%
HILICPOS	D9-Betaine	127.1427	7.25	13%
HILICPOS	D9-Butyrobetaine	155.174	7.83	6%
HILICPOS	D9-Choline	113.1635	5.18	6%
HILICPOS	D9-Crotonobetaine	153.1584	7.86	9%
HILICPOS	D9-TMAO	85.1322	5.57	8%
HILICPOS	Val-Tyr-Val	380.218	6.95	24%

## Supplementary Materials

The following are available online at https://www.mdpi.com/2218-1989/9/5/101/s1, Table S1: Phenotype details; Table S2: Phenotype data for knockouts; Table S3: WCMC—GCMS annotation database; Table S4: WCMC—HILIC annotation database (ESI POS); Table S5: WCMC—HILIC annotation database (ESI NEG); Table S6: WCMC—CSH annotation database (ESI POS); Table S7: WCMC—CSH annotation database (ESI NEG); Table S8: WCMC—Targeted metabolomics database (Bile Acids and Steroids); Table S9: WCMC—Targeted metabolomics database (Oxylipins); Table S10 Sample and file name mapping; Table S11: Raw metabolomics dataset for mouse knockouts; Table S12: Filtered metabolomics dataset; Table S13: Data Dictionary; Table S14: Data Matrix; Table S15: Sample metadata; Table S16: Reagents and material used; Table S17: Analysis sequences for all assays; Table S18: Internal standards for LCMS assays; Figure S1: Total ion chromatogram plots for LC-MS data of quality control samples.

## Data Set

The dataset is available at the MetabolomicsWorkbench repository (accession ID: ST001154)

## Data Set License

License under which the data set is made available (CC0).
