# Supplementary material for: A Comprehensive Plasma Metabolomics Dataset for a Cohort of Mouse Knockouts within the International Mouse Phenotyping Consortium
Source: Metabolites. 2019 May 22;9(5):101. doi: 10.3390/metabo9050101 (PMC6571919; doi:10.3390/metabo9050101)
Supplement: Supplementary file 1 [file metabolites-09-00101-s001.zip › metabolites-505978-SI/Supplementary figure s1.pptx]

## Slide 1
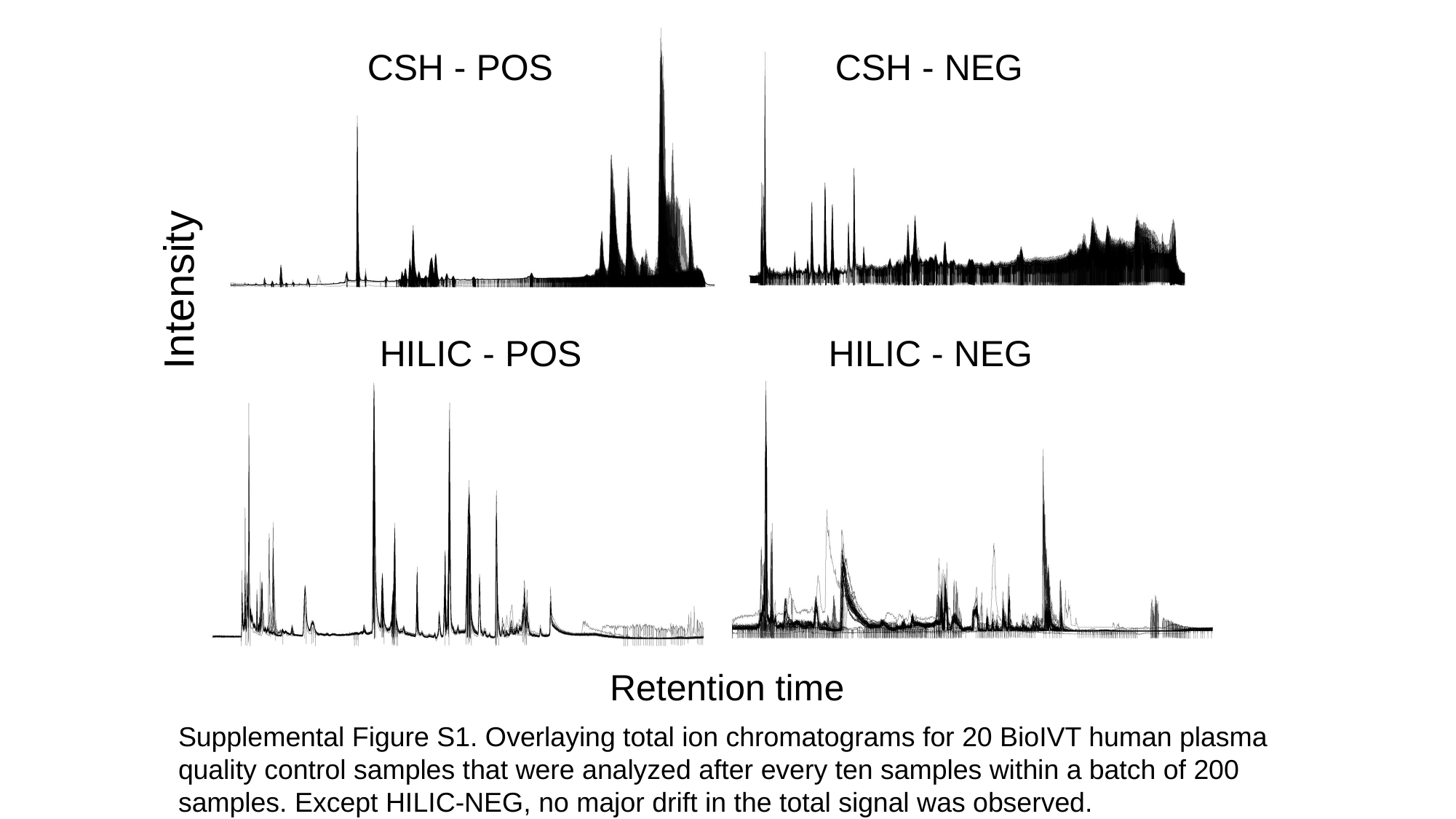

CSH - POS
CSH - NEG
Intensity
HILIC - POS
HILIC - NEG
Retention time
Supplemental Figure S1. Overlaying total ion chromatograms for 20 BioIVT human plasma quality control samples that were analyzed after every ten samples within a batch of 200 samples. Except HILIC-NEG, no major drift in the total signal was observed.
